# A third of all abstracts from the 2009 and 2010 Danish Emergency Medicine Conferences have been published as full-text articles: a retrospective study

**DOI:** 10.1186/1757-7241-21-S2-A15

**Published:** 2013-09-09

**Authors:** Mikkel Brabrand, Dan Brun Petersen, Lars Folkestad, Peter Hallas

**Affiliations:** 1 Danish Society for Emergency Medicine, Denmark; 2 Department of Medicine, Sydvestjysk Sygehus Esbjerg, Denmark; 3 Department of Endocrinology, Sydvestjysk Sygehus Esbjerg, Denmark

## Background

Many authors initially present study data as an abstract at a medical conference. Later on, after initial presentation, the study should ideally be presented as a full-text article in a peer-reviewed journal, regardless if the findings were positive or negative. Previous studies have shown that approximately a third of abstracts presented at Emergency Medicine conferences are published as peer-reviewed articles. We set out to establish the proportion of abstracts presented at the Danish Emergency Medicine Conferences (DEMC) in 2009 and 2010 that were published as articles in peer-reviewed journals.

## Methods

This is a retrospective study using the lists of accepted abstracts from the 2009 and 2010 DEMC published in the Scandinavian Journal of Trauma, Resuscitation and Emergency Medicine. As a sub analysis, we included the abstracts from the 2011 DEMC, but as this was held less than 18 months ago, we excluded the numbers from analyses. We manually searched PubMed using the names of the authors and extracts of the titles of the abstracts up to January 2013. Data will be presented descriptively and differences between proportions tested using Chi-square test.

## Results

From the 2009 DEMC, 19 abstracts were published. Of these, six (31.6 %) had been published as full-text articles in peer-reviewed journals. As for the 2010 DEMC, 44 abstracts were published from the conference and 12 (27.3 %) of these had been published as full-text articles, p = 0.73. Six of all the published abstracts had been presented as oral presentation, and four of these (66.7 %) had been published while 14 (24.6 %) of the 57 abstracts presented as poster presentations had been published as full-text articles, p = 0.03. From the 2011 DEMC, 55 abstracts were published and six (10.9 %, none of which were oral presentations) have later been published as full-text peer-reviewed articles.

## Conclusion

Approximately one-third of abstracts published from the 2009 and 2010 Danish Emergency Medicine Conferences have been published as full-text articles in peer-reviewed journals by January 2013. Significantly more abstracts presented as oral presentations have been published. These numbers are similar to larger international Emergency Medicine conferences.

